# A Homozygous *AKNA* Frameshift Variant Is Associated with Microcephaly in a Pakistani Family

**DOI:** 10.3390/genes12101494

**Published:** 2021-09-24

**Authors:** Syeda Seema Waseem, Abubakar Moawia, Birgit Budde, Muhammad Tariq, Ayaz Khan, Zafar Ali, Sheraz Khan, Maria Iqbal, Naveed Altaf Malik, Saif ul Haque, Janine Altmüller, Holger Thiele, Muhammad Sajid Hussain, Sebahattin Cirak, Shahid Mahmood Baig, Peter Nürnberg

**Affiliations:** 1 Cologne Center for Genomics (CCG), Faculty of Medicine, University Hospital Cologne, University of Cologne, 50931 Cologne, Germany; syeda_sam@hotmail.com (S.S.W.); a.bakar@live.com (A.M.); b.budde@uni-koeln.de (B.B.); maria00415@yahoo.com (M.I.); janine.altmueller@bih-charite.de (J.A.); hthiele@uni-koeln.de (H.T.); mhussain@uni-koeln.de (M.S.H.); 2 Center for Biochemistry, Medical Faculty, University of Cologne, 50931 Cologne, Germany; 3 Center for Molecular Medicine Cologne (CMMC), Faculty of Medicine, University Hospital Cologne, University of Cologne, 50931 Cologne, Germany; sebahattin.cirak@uk-koeln.de; 4 Department of Pediatrics, Faculty of Medicine, University Hospital Cologne, University of Cologne, 50931 Cologne, Germany; 5 Human Molecular Genetics Laboratory, Health Biotechnology Division, National Institute for Biotechnology and Genetic Engineering (NIBGE), PIEAS, Faisalabad 38000, Pakistan; tariqpalai@gmail.com (M.T.); ayazgenetics@gmail.com (A.K.); sherazkhanhu@gmail.com (S.K.); naveednibge@gmail.com (N.A.M.); shahid_baig2002@yahoo.com (S.M.B.); 6 Center for Biotechnology and Microbiology, University of Swat, Swat 19130, Pakistan; zafaralibiotech@gmail.com; 7 Nuclear Medicine, Oncology and Radiotherapy Institute (NORI), PAEC, Islamabad 44000, Pakistan; drsaifulhaque@yahoo.com; 8 Pakistan Science Foundation (PSF), 1-Constitution Avenue, G-5/2, Islamabad 44000, Pakistan; 9 Department of Biological and Biomedical Sciences, Aga Khan University, Karachi 74000, Pakistan

**Keywords:** autosomal recessive primary microcephaly (MCPH), *AKNA*, whole-exome sequencing (WES), linkage/haplotype analysis, cerebral cortex

## Abstract

Primary microcephaly (MCPH) is a prenatal condition of small brain size with a varying degree of intellectual disability. It is a heterogeneous genetic disorder with 28 associated genes reported so far. Most of these genes encode centrosomal proteins. Recently, AKNA was recognized as a novel centrosomal protein that regulates neurogenesis via microtubule organization, making *AKNA* a likely candidate gene for MCPH. Using linkage analysis and whole-exome sequencing, we found a frameshift variant in exon 12 of *AKNA* (NM_030767.4: c.2737delG) that cosegregates with microcephaly, mild intellectual disability and speech impairment in a consanguineous family from Pakistan. This variant is predicted to result in a protein with a truncated C-terminus (p.(Glu913Argfs*42)), which has been shown to be indispensable to AKNA’s localization to the centrosome and a normal brain development. Moreover, the amino acid sequence is altered from the beginning of the second of the two PEST domains, which are rich in proline (P), glutamic acid (E), serine (S), and threonine (T) and common to rapidly degraded proteins. An impaired function of the PEST domains may affect the intracellular half-life of the protein. Our genetic findings compellingly substantiate the predicted candidacy, based on its newly ascribed functional features, of the multifaceted protein AKNA for association with MCPH.

## 1. Introduction

Autosomal recessive primary microcephaly (MCPH, for “microcephaly primary hereditary”) is a rare neurodevelopmental disorder characterized by a reduced head circumference (HC) with more than three standard deviations (SD) below the mean for the same age and sex. MCPH is congenital with a varying degree of cognitive impairments in addition to small brain size. The cerebral cortex size of a patient is reduced in a strikingly selective manner affecting volume and surface area of all cortical regions except for the hippocampus and surrounding medial temporal structures [1]. Primary microcephaly is a genetically heterogeneous disorder that is considered as a model disease for impaired forebrain neocortex development [2,3,4]. The prevalence of recessive MCPH has been estimated at 1/100,000 births in Western countries, and is approximately ten times higher in the Asian and Arab countries where cousin marriages are common [5,6].

To date, 28 genes have been reported for MCPH with *ASPM* (MCPH5) and *WDR62* (MCPH2) as the most frequently mutated genes in approximately 50% and 14% of all studied families, respectively [7,8,9,10]. Many of these MCPH genes encode centrosomal proteins that are essentially needed in centriole biogenesis [11]. Others are known to be implicated in diverse mechanistic pathways such as DNA replication and repair, cytokinesis, kinetochore function, Wnt signaling, transmembrane or intracellular transport [12]. Thus, it suggests a diverse involvement of molecular and cellular mechanisms in controlling the cerebral cortex size during brain development. More detailed investigations on this rare syndrome can divulge the common molecular pathways necessary for the maintenance and regulation of neural progenitor cells (NPCs) that are responsible for determining the cerebral cortex size and human brain evolution.

The microtubule organization protein AKNA was first described as KIAA1968, comprising a DNA-binding motif (called AT-hook) that specifically binds to AT-rich regions to regulate transcription of cellular immune response genes [13]. Reports on a knockout mouse model and truncating *AKNA* variants in dogs and humans have pointed to an inflammatory lung pathology or primary ciliary dyskinesia-like clinical picture with recurrent sinopulmonary infections as a major phenotypic consequence [14,15,16]. Recently, AKNA was found to have a role during prenatal stages of brain development in mice and in human cerebral organoids derived from human induced pluripotent stem cells (hiPSCs) [17]. The centrosomal localization of AKNA allows this protein to perform its function during brain development efficiently with the C-terminal part of AKNA being essential for its localization to the centrosome.

Here, we provide further evidence on its recently uncovered role in the regulation of neurogenesis by linking *AKNA* to MCPH in a consanguineous family from Pakistan with three affected children with consistently reduced head circumference and intellectual disability as the most prominent features.

## 2. Materials and Methods

### 2.1. Clinical Manifestations

The family included in this study was recruited from the Pashtun population in the North-West of Pakistan (Figure S1). The ethics committees of the National Institute for Biotechnology and Genetic Engineering in Faisalabad, Pakistan, and the University Hospital Cologne approved the study. Written informed consent was obtained from all participants before inclusion into this study. Clinical investigation was performed locally including interviews with first- and second-degree relatives. For the index patient, MRI scans of the head and chest CT scans were done at the district hospital. Genomic DNA was extracted from peripheral blood leukocytes using the Blood and Cell Culture DNA Midi Kit from QIAGEN (Hilden, NRW, Germany).

### 2.2. Linkage Analysis

The three affected siblings were genotyped using the HumanCoreExome 24 v.1.1 BeadArray from Illumina (San Diego, CA, USA). Subsequent data handling was performed using the graphical user interface ALOHOMORA v. 0.33.1 [18]. Relationship errors were identified using the program Graphical Relationship Representation [19]. The program PedCheck v. 1.1 was applied to find Mendelian errors and data of single nucleotide polymorphism (SNP) markers, with such errors removed from the data set [20]. Non-Mendelian errors were identified using the program MERLIN v. 1.1.2 and unlikely genotypes for related samples were deleted [21]. Linkage analysis was performed assuming autosomal recessive inheritance and a disease allele frequency of 0.0001. Multipoint LOD (logarithm of the odds) scores were calculated using MERLIN [21]. Haplotypes were generated also with MERLIN and displayed graphically using the program HaploPainter v. 1.046 [22].

### 2.3. Next Generation Sequencing

Initially, proband IV-2 tested negative for the Pakistani founder mutation (NM_018136.4: c.3978G > A) in exon 17 of *ASPM* [23]. Thereafter, a customized gene panel (Agilent SureSelectXT) was sequenced, consisting of 86 candidate genes for primary or syndromic microcephaly [24], but no potentially disease-causing variant was identified. Finally, we used the SureSelectXT Human All Exon V6 enrichment kit from Agilent Technologies (Santa Clara, CA, USA) to perform whole-exome sequencing (WES) on DNA of individual IV-3. Samples were sequenced on an Illumina NovaSeq 6000 (San Diego, CA, USA) with 2 × 100 bp paired-end reads. The experimental method and data handling were performed as described previously [25]. Only sequencing reads with a minimum coverage of ≥10× were considered for further analyses.

### 2.4. In Silico Analyses of Identified Variants

For mapping and annotation of variants, the GRCh37/hg19 genome assembly was used as a reference. Variant calling files were created using our VARBANK pipeline (https://varbank.ccg.uni-koeln.de, accessed on 19 September 2021). As a start, the default filter criteria of VARBANK were adjusted to detect rare homozygous variants with an allele read frequency between 75–100%, thereafter we also filtered for compound heterozygous and X-linked variants and excluded all variants with a high allele frequency and/or without any disease-related function (Table S1).

Data scrutiny was focused on single-nucleotide variants (SNVs) and insertions or deletions (InDels) that could have a deleterious effect on the protein structure or function. Variants were ranked for minor allele frequency <0.001 in public databases such as gnomAD (https://gnomad.broadinstitute.org, accessed on 19 September 2021), public exome variant server (https://evs.gs.washington.edu/EVS/, accessed on 19 September 2021), 1000 genome (http://browser.1000genomes.org, accessed on 19 September 2021), dbSNP151 (https://www.ncbi.nlm.nih.gov/snp/, accessed on 19 September 2021), Iranome (http://www.iranome.ir/, accessed on 19 September 2021) and the Greater Middle Eastern Variome (http://igm.ucsd.edu/gme/index.php, accessed on 19 September 2021). VARBANK 2.0 was used for the variant feature search as it integrates data from a significant number of publicly available data resources. With the help of VARBANK 2.0, functional effect predictions were made for the filtered variants using criteria like CADD Phred score >20 (http://cadd.gs.washington.edu/, accessed on 19 September 2021), variant effect predictor (https://www.ensembl.org/Tools/VEP, accessed on 19 September 2021) and Mutation Taster definition as disease-causing (https://www.genecascade.org/MutationTaster2021/, accessed on 19 September 2021). Evolutionary conservation of the altered amino acids was determined by multiple sequence alignment (MSA) using NCBI and Clustal Omega (https://www.ebi.ac.uk/Tools/msa/clustalo/, accessed on 19 September 2021).

### 2.5. Sanger Sequencing

Sanger sequencing was performed on an ABI3730 Genetic Analyzer from Applied Biosystems (Waltham, Massachusetts, USA) for the verification of sequence variants identified by next-generation sequencing (NGS) and segregation analysis. PCR conditions and primers that were used to amplify the specific regions of *AKNA* are available upon request.

## 3. Results

**Figure 1:**
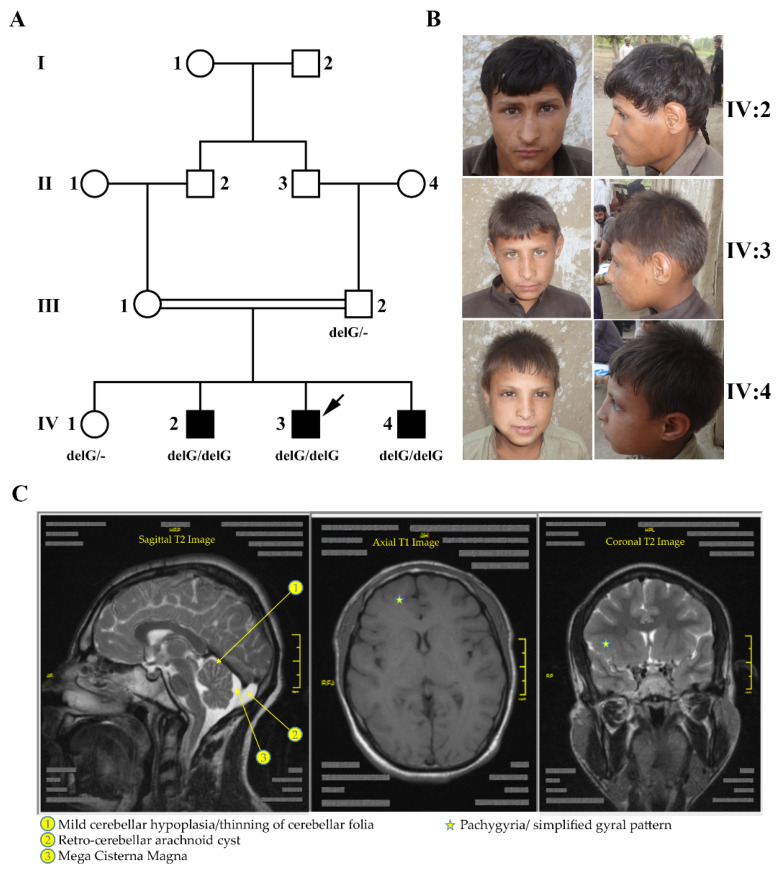
Ascertainment of a Pakistani family with primary microcephaly. (**A**) Pedigree of a consanguineous family afflicted with autosomal recessive primary microcephaly, recruited from Northern Pakistan. The arrow points to the person whose DNA was subjected to WES. Genotypes for *AKNA* variant c.2737delG are indicated below the symbols of the tested family members. (**B**) Photographs of the three affected siblings in the fourth generation who exhibit microcephaly along with intellectual disability and mild speech/hearing deficit. (**C**) MRI images of the brain of individual IV-3. In addition to the reduced cerebral cortex, mild cerebellar abnormalities, pachygyria and a frontal simplified gyral pattern are visible.

We ascertained a consanguineous MCPH family with three affected siblings from a city in the northwest of Pakistan (Figure 1, Figure S1). Using standard charts of head circumference and by reviewing medical records, three brothers born to consanguineous parents with first-degree relation were diagnosed with microcephaly (head circumference of −7 SD and below) accompanied by signs compatible with mild manifestation of primary ciliary dyskinesia (PCD) such as swollen nasal mucosae, nasal voice and recurrent fever due to frequent sinusitis (Table 1 and Figure S2). A computed tomography (CT) scan of the chest of the index patient did not show any abnormalities of the lung (Figure S3).

**Figure 2:**
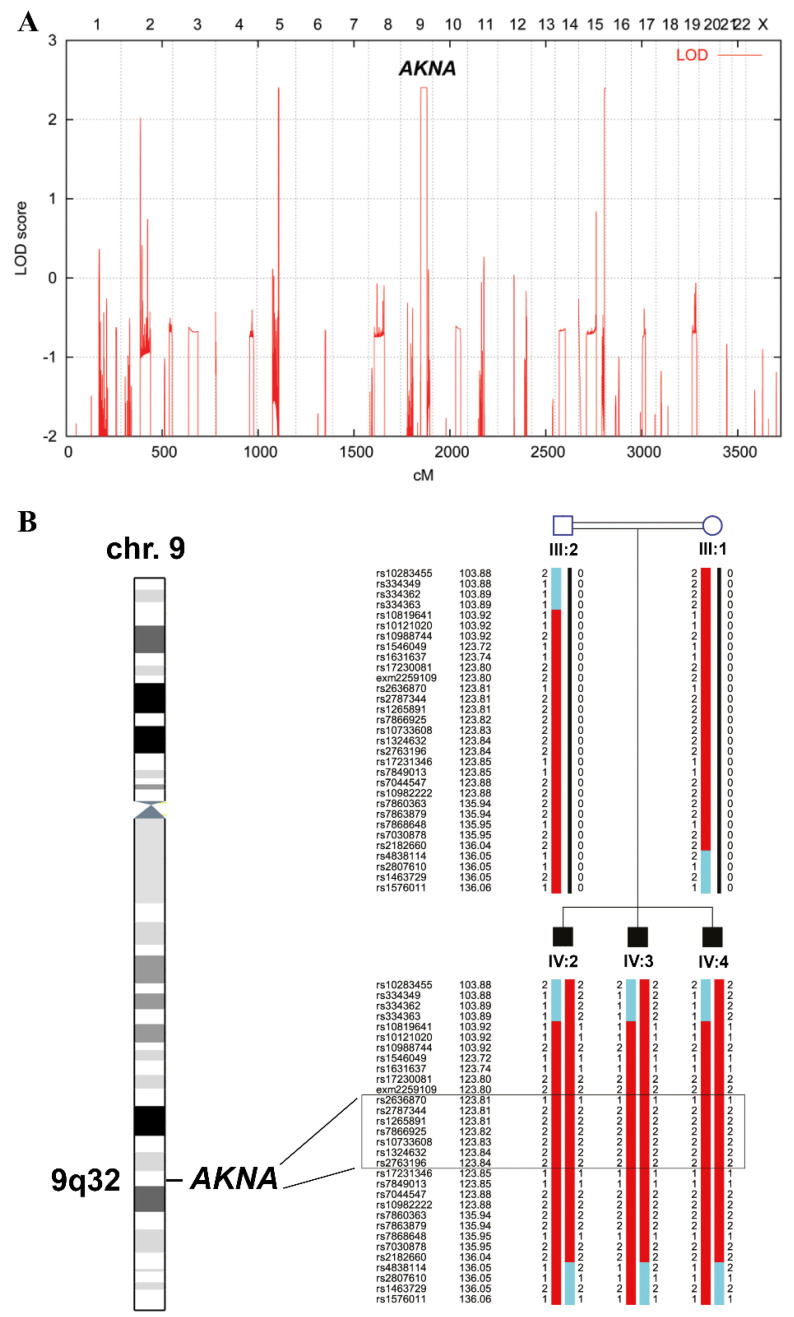
Mapping of a new locus for MCPH on chromosome 9. (**A**) Genome-wide LOD (logarithm of the odds) scores generated from SNP array data. A 25 K panel of equally distributed markers with a minor allele frequency higher than 0.10 was used. Scores are plotted over genetic distance across the genome according to the deCODE map. Chromosomes are concatenated from p-ter to q-ter from left to right. The highest scores were obtained for markers on chromosomes 5, 9 and 15 with the linkage signal on chromosome 9 at cytoband 9q32 representing by far the longest run of homozygosity. The LOD score was equal to 2.4, which is the theoretical maximum for this family assuming full informativity of the marker set. (**B**) Haplotypes of the linkage region on chromosome 9 including *AKNA*. The size of the homozygous region is 24.9 Mb. The heterozygous markers flanking this interval are rs334363 (101,928,702 bp) at 103.89 cM and rs4838114 (126,875,893 bp) at 136.05 cM, referring to build GRCh37.p13. SNP markers of the *AKNA* region are boxed.

Genome-wide linkage analysis resulted in three peaks—on chromosomes five, nine and 15—reaching the theoretical maximum LOD score of 2.4 for the recorded pedigree (Figure 2A). Since no known MCPH genes were located in any of the linkage regions, we searched for new candidate genes and together with the WES data identified *AKNA* on chromosome nine as the most promising one. It is located within the largest homozygous region shared between the three affected brothers. Figure 2B delineates the position of *AKNA* within this interval, which is limited by SNP markers rs334363 (103.89 cM, 101,928,702 bp in genome build GRCh37) and rs4838114 (136.05 cM, 126,875,893 bp).

**Figure 3:**
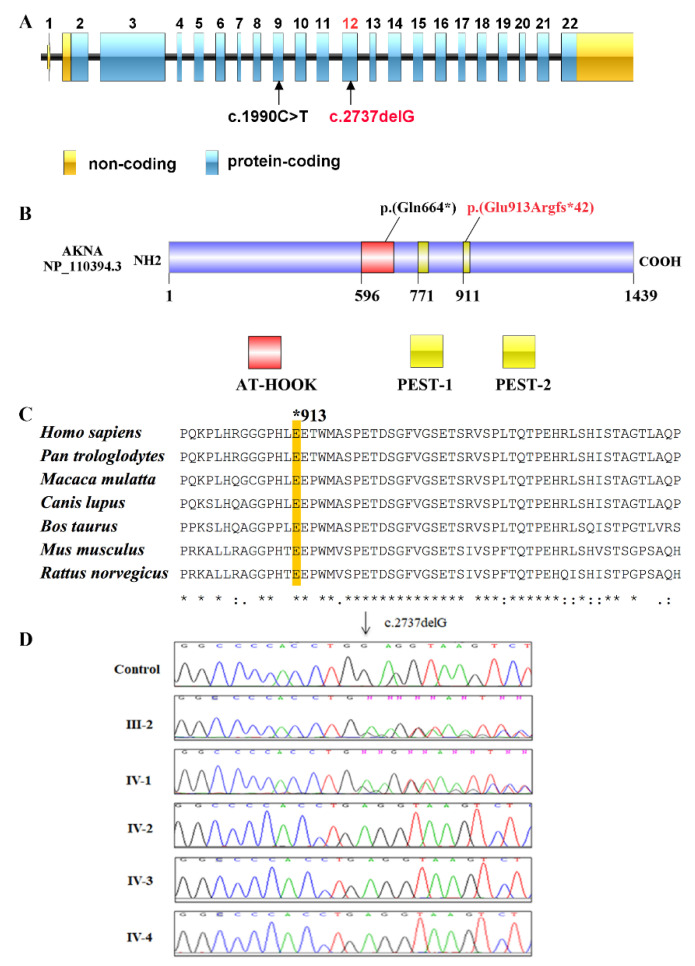
Identification of a homozygous variant in *AKNA*. (**A**) Schematic representation of the *AKNA* gene structure. All exons (boxes) are drawn to scale and separated by lines of arbitrary length to delineate the introns (Illustrator for Biological Sequences (IBS) v 1.0.1). The novel variant was identified in exon 12. Previously, a variant in exon 9 had been found and tentatively associated with PCD [16]. Blue boxes indicate the coding part while yellow boxes represent the untranslated regions at the 3′ and 5′ ends. (**B**) Schematic representation of the AKNA protein structure comprising 1439 amino acids. Domains are highlighted by the specified color code; the yellow color is presenting the proline (P), glutamic acid (E), serine (S), and threonine (T) (PEST) domains. The novel truncating variant p.(Glu913Argfs*42) is located at the start of the second PEST domain. The red color marks the AT-hook-containing transcription factor domain (AT-hook). A variant in that domain, p.(Gln664*), has recently been reported to be associated with PCD and to cause sinopulmonary infections [16]. (**C**) Multiple sequence alignment of human AKNA homologous proteins. Most of the miscoded amino acids in the truncated protein at and after position Glu913 are strictly conserved in vertebrates. The alignment was performed with Clustal Omega. Protein accession numbers from NCBI are as follows: NP 110394.3_*H. sapiens*, XP_003312312.2_*P. troglodytes*, XP_001096711.2_*M. mulatta*, XP_538809.3_*C. lupus*, XP_002689997.3_*B. taurus*, NP_001038979.1_*M. musculus*, NP_001102138.1_*R. norvegicus.* (**D**) Sanger sequence electropherograms of all available family members and a control to document co-segregation between the congenital microcephaly phenotype and the recessive disease allele. As expected, all patients are homozygous for NM_030767.4:c.2737delG in exon 12 of *AKNA* whereas the father and the normal sibling are heterozygous carriers of the mutant allele.

After exclusion of all known MCPH-associated genes by gene panel sequencing, we subjected DNA of the affected sibling IV-3 to WES and identified a homozygous frameshift variant in *AKNA* (AT-hook transcription factor)—NM_030767.4:c.2737delG, p.(Glu913Argfs*42)—as the most likely cause of MCPH in this family. This variant is located in exon 12 of *AKNA* (Figure 3A) and leads to a premature termination codon (PTC) after a frameshift sequence of 41 amino acids following the codon affected by the single base-pair deletion (Figure 3B). Thus, the variant is predicted to generate a severely truncated protein. Definitely, its emerging role in cortical neurogenesis during brain development [17] gave direction to our choice to select the gene coding for the centrosomal protein AKNA, but variants in other genes could be all excluded based on their allele frequencies in several public databases and the absence of any plausible functional relationship to the disease phenotype. When excluding the other variants we also considered compound-heterozygous inheritance patterns and X-chromosomal variants (Table S1).

The out-of-frame deletion NM_030767.4:c.2737delG, p.(Glu913Argfs*42) is predicted to be deleterious by Mutation Taster as it interrupts the normal reading frame and results in premature termination of translation. The first amino acid affected by miscoding at position 913 as well as many of the 40 following ones, until the PTC is reached at position 954, are highly conserved in vertebrates (Figure 3C). Of note, the introduction of a PTC due to the frameshift variant is likely to induce nonsense-mediated decay (NMD) of the mutated mRNA as predicted by NMD Esc predictor, which may reduce the relative abundance of the mutated transcripts and also the corresponding translated protein molecules in the cells. Moreover, the *AKNA* variant identified in this study is not found in a homozygous state in databases like dbSNP151, 1000 Genomes (build 20110521), the public Exome Variant Server, NHLBI Exome Sequencing Project (ESP), Seattle (build ESP6500) and BRAVO. It is also completely absent from our in-house database (Cologne Center for Genomics) with several thousand exomes. Interestingly, the Genome Aggregation Database (gnomAD v2.1.1) contains nine heterozygous carriers of the variant allele (rs368704395) exclusively in the African population sample (Table S1). Notably, *AKNA* variants are neither present in homozygous nor heterozygous state in the Greater Middle Eastern Variome Project and Iranome database. Our family is of Asian ethnicity with a consanguineous background and the identified *AKNA* variant cosegregates with the phenotype in all available family members in an autosomal recessive manner as revealed by Sanger sequencing. All affected siblings are homozygous whereas the father and the normal sibling are heterozygous for the *AKNA* variant (Figure 3D).

## 4. Discussion

Our genetic analysis of an MCPH family with three affected siblings from Pakistan resulted in the identification of a frameshift variant in *AKNA* (MIM*605729) as the only plausible cause of the disease. Recently, AKNA was ascribed a new function as a centrosomal protein with a key role in the regulation of neurogenesis [17]. Hence, our finding is in keeping with the observation that most MCPH-associated genes are coding for centrosomal proteins [4,25,26,27]. AKNA localizes on subdistal appendages of the mother centriole during interphase. It is also found at the proximal ends of centrioles and along microtubules. AKNA dissociates from the centrosomes during M-phase without proteolytic degradation and reassembles at the centrosomes during late telophase and early G1 phase. Dissociation and reassembly is regulated by phosphorylation [17].

AKNA is a potent regulator of the centrosome, controlling its capacity of microtubule organization specifically in differentiating neural stem cells (NSCs) [17]. The protein also boosts the growth of microtubules by recruiting components of the *γ*-tubulin ring complex like TUBG and GCP4 [28]. These characteristics of AKNA make this protein highly compelling in terms of delamination and subventricular zone (SVZ) generation during brain development [29]. The C-terminal part of AKNA controls its localization on the centrosome to regulate corticogenesis efficiently, as it was shown by Camargo Ortega et al. 2019 [17] that an AKNA protein lacking the C-terminal (324 amino acids) was dispersed in the cytoplasm, missing its localization on the centrosome. Notably, the loss-of-function variant of *AKNA* investigated in this study results in a truncation of the C-terminus, likely deteriorating its localization to the centrosome and leading to an impaired cortical neurogenesis. Astonishingly, the C-terminal region was overlooked in earlier studies on AKNA, resulting in a focus on its role in immunity and inflammation further on [13]. It is difficult to retrace why Siddiqa et al. 2001 [13] did not use the complete clone that includes the sequence for the C-terminal part of AKNA in their studies. As a matter of fact, by using clones with missing sequences they did not recognize AKNA’s link to centrosomes as an important feature of this protein [17].

*AKNA* maps to the fragile FRA9E region of human chromosome 9q32. The gene locus spans 61 kb and comprises 22 exons. There are at least nine distinct transcripts and the largest one codes for a protein of 155 kDa with 1439 amino acids (Figure 3B). The different transcripts seem to originate from differential promoter usage and alternative splicing and may encode overlapping protein isoforms [30]. Another versatility in gene regulation in higher eukaryotes is achieved by polyadenylation, which can control mRNA stability and translation rates [31]. Most of the *AKNA* transcripts use a polyadenylation site in exon 22 [30]. Notably, the region harboring the identified variant is present in most of the transcripts. *AKNA* transcript diversity may be instrumental in attaining its functional diversity as it acts as a neurogenesis regulator in the cerebral cortex during brain development as well as a transcription factor in lymphoid organs [13,17].

Very recently, a homozygous nonsense variant in *AKNA* has been reported in a family with a PCD-like phenotype [16], however, the authors did not show abnormally beating motile cilia, thus the chronic pulmonary infections and alteration of mucosal clearance with the PCD-like phenotype might be secondary due to other functions of ANKA in mucosal homoeostasis. The variant that they identified, NM_030767:c.1990C > T, p.(Gln664*), is predicted to result in a PTC in the AT-hook domain of AKNA and seems to interfere more prominently with the transcription factor function of AKNA rather than the regulation of microtubule organization during neurogenesis. In contrast to its suggested association with PCD, AKNA does not seem to play a role in ciliogenesis, as neither loss nor gain of function have a relevant effect on ciliogenesis, neither on the ciliary structure nor on their number [15,17]. In our patients, we did not observe chronic pulmonary infections despite clear features of primary microcephaly. Nevertheless, the PCD-like phenotype in our cases might be rather hypomorphic as we also noticed swollen mucosae of nasal sinuses in the MRI scans of our patients (see Figure S2). PCD is usually described as the clinical syndrome of dysfunctional motile cilia [32]. Motile cilia are visible only in specific cell types such as the respiratory epithelium, ependymal lining of brain ventricles, embryonic node, and oviducts and in sperm cells [33]. Motile cilia are dynamic in their structure and function. Dysfunction of motile cilia in the respiratory system manifests as chest infections, sinusitis and non-seasonal rhinorrhea, whereas impaired function in sperm cells may lead to male infertility. In addition, abnormal ciliary beating in the ependymal lining of brain ventricles in the mice results in hydrocephalus. Even though hydrocephalus is only sporadically related to PCD in patients, it is very common in mouse models [34]. It remains true that identifying PCD with variable overlapping clinical phenotypes poses a diagnostic challenge.

Targeted deletion of exons 19–21 or disruption of exon three in *AKNA* knockout mice resulted in feeble, small size mice with severe alveolar damage and sudden neonatal death [14]. The authors did not mention any brain phenotype in these animals. Camargo Ortega et al., however, documented an indispensable role of *AKNA* in controlling neurogenesis in the mouse cerebral cortex via microtubule organization [17]. In the developing cerebral cortex, *AKNA* shows its highest expression signal at E14 in differentiating neural stem cells (NSCs) in the SVZ and low signals at E18 in the cortical plate where differentiating neurons reside [35]. Low levels of *AKNA* after E14 are highly essential for the exit of differentiating basal progenitors from the intermediate zone (IZ) to the cortical plate (CP) [17]. Loss of function of *AKNA* in cerebral cortical slices of mice during brain development drastically impairs NSCs’ delamination, ultimately resulting in the accumulation of more NSCs in the ventricular zone but with fewer neurons [17]. Inversely, *AKNA* overexpression promotes fast delamination, eventually leading to more neurons that populate in the SVZ and are unable to reach the CP [17]. Human cerebral organoids generated from hiPSCs also show elevated expression of AKNA in the SVZ during cortex formation; and irregular patterns of *AKNA* expression in human NPCs derived from hiPSCs reveal defects in cortical neurogenesis affecting normal brain development [17]. Thus, the timely and controlled expression of *AKNA* at different embryonic stages is mandatory for the normal expansion of the cerebral cortex. This suggests the possibility that the identified loss-of-function variant in our study could affect the timely expression of *AKNA*, thus disturbing the maintenance of the NPC pool and resulting in fewer neurons with thinning of the cerebral cortex and finally microcephaly.

AKNA possesses two PEST domains—protein motifs which are largely enriched in proline (P), glutamic acid (E), serine (S), and threonine (T) residues. They are needed for protein cleavage and are common in regulatory proteins with rapid turnover [13]. Protein degradation has an essential role in controlling a protein’s function and PEST domains fulfill a central role in this regard [36,37,38]. PEST-dependent proteolysis is a mechanism which is used to control the function of transient or maturing proteins [39]. PEST-dependent cleavage of AKNA is crucially important to perform its function efficiently in DNA binding [30]. Intriguingly, the string of 41 miscoded amino acids (p.(Glu913Argfs*42)) in our patients nearly completely overwrites the second PEST domain (amino acid residues 911–932), which may hinder its function in protein cleavage at a specific developmental stage of the brain, thereby drastically affecting the proliferation and differentiation of the NPC pool and in turn resulting in microcephaly.

Phenotypical disparity has been previously described in many genetic conditions for the genes encoding centrosomal proteins [40]. For example, variants in *CEP290* and *CEP135* have been reported to lead to divergent disorders with different phenotypes [25,41,42,43,44]. Another reason for the divergent phenotype of *AKNA* variants might be explained by a dosage-dependent mechanism in which a late truncation of the AKNA protein product is still adequate to provide sufficient residual activity in humans to allow a nearly normal ciliary function and, perhaps, a less severe phenotype of PCD. Finally, further variants in the individual genomes may modify the phenotypic consequences of the *AKNA* variants.

In summary, we report on a novel primary microcephaly associated gene—*AKNA*—revealed by a homozygous truncating variant found in a consanguineous Pakistani family with three affected siblings. It seems plausible that primary microcephaly in our family is the consequence of the lost C-terminus and impaired second PEST domain of the truncated AKNA protein. Together with the functional data published by others [17], our finding highlights a decisive role of centrosomal proteins in the timely regulation and maintenance of the NPC pool during prenatal stages of brain development. Future cases with *AKNA* variants will be needed to discern the phenotypic discrepancies and will provide important insights into genotype–phenotype correlations and perturbed pathways.

## 5. Conclusions

The clinical and genetic data presented in our study provide clear indications for the presence of autosomal recessive primary microcephaly (MCPH) along with mild manifestation of PCD-like features in a consanguineous Pakistani family with a pathogenic *AKNA* variant, but it is left to future functional analyses to shed light on the deep-down compendium of complex regularity networks for *AKNA*. Our results are unique because this is the first study presenting *AKNA* as a likely candidate gene for MCPH in keeping with the results of its profound functional characterization during early embryonic stages of the mouse brain development and its localization to the centrosome presented in a recent study by others [17]. Based on our data, we would recommend geneticists and researchers to consider thoroughly the multifaceted roles of genes and proteins when elucidating rare genetic syndromes.

## Figures and Tables

**Table 1:** Survey of clinical investigations.

Patient Characteristics		Patients with a Novel Homozygous Frameshift Variant in *AKNA* (c.2737delG)		
Patient ID		IV-2	IV-3	IV-4
Origin		Pakistani	Pakistani	Pakistani
Gender		Male	Male	Male
Age		15 years	9 years	6 years
Pregnancy		Normal	Normal	Normal
Head circumference (HC)		43 cm (−7.5 SD)	43 cm (−7.0 SD)	42 cm (−7.5 SD)
Hypotonia		No	No	No
Delay of motor milestones		Not reported	Not reported	Not reported
Seizures		No	No	No
Cardiac problems		No	No	No
Facial anomalies		No	No	No
Ophthalmologic findings		No	No	No
Hearing impairment		Yes (can hear only loud sounds)	Yes (can hear only loud sounds)	Yes (can hear only loud sounds)
Gait abnormality		No	No	No
Speech impairment		Yes (only few words, no sentences)	Yes (only few words, no sentences)	Yes (only few words, no sentences)
Cognitive skills (problem solving, judgement power)		Not age appropriate	Not age appropriate	Not age appropriate
Self-feeding		Yes	Yes	Yes
Self-clothing		Yes	Yes	Yes
Ataxia		No	No	No
Learning disability		Yes (no schooling at all)	Yes (no schooling at all)	Yes (no schooling at all)
Limb/trunk deformities		No	No	No
Muscle function (muscle tone, strength, endurance)		Normal	Normal	Normal
Nasal voice		Yes	Yes	Yes
Recurrent fever		Yes	Yes	Yes
MRI brain	Age at scan	NR	15 years	NR
	Simplified gyral pattern	NR	Yes	NR
	Microcephaly	NR	Yes	NR
	Thin filea	NR	Yes	NR
	Mega cisterna magna	NR	Yes	NR
	Thick nasal mucosa	NR	Yes	NR
	Brain stem	NR	Normal	NR
	Others	NR	Normal	NR
Chest CT	Lung	NR	Normal	NR

NR: No Record; HC: Head Circumference; SD: Standard Deviation.

## Data Availability

The data presented in this study are available on request from the corresponding author. The data are not publicly available due to privacy issues.

## Supplementary Materials

The following are available online at www.mdpi.com/article/10.3390/genes12101494/s1, Figure S1: Map displaying the home area of the MCPH family, Figure S2: MRI images of individual IV-3 to document signs of PCD, Figure S3: Thoracic CT scan of individual IV-3, Table S1: Highest ranking homozygous, X-linked and compound heterozygous variants detected by WES.
