# Supplementary material for: A Homozygous AKNA Frameshift Variant Is Associated with Microcephaly in a Pakistani Family
Source: Genes (Basel). 2021 Sep 24;12(10):1494. doi: 10.3390/genes12101494 (PMC8535656; doi:10.3390/genes12101494)
Supplement: Supplementary file 1 [file genes-12-01494-s001.zip › genes-1371335-supplementaryy.pdf]

Supplementary Material

to

A homozygous *AKNA* frameshift variant is associated with primary microcephaly in a Pakistani family

by

Waseem et al.

The supporting information contains three supplementary figures and one supplementary table.

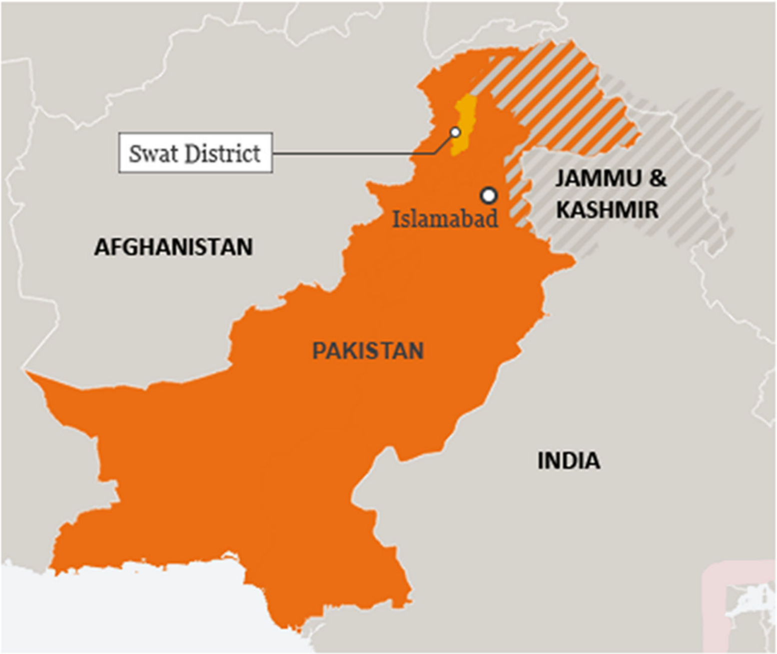

Figure S1. Map displaying the home area of the MCPH family. It belongs to the Pashtun population of North-West Pakistan (Adopted from: https://www.dw.com/en/women-defy-local-traditions-in-pakistans-swat-valley/a-38609291).

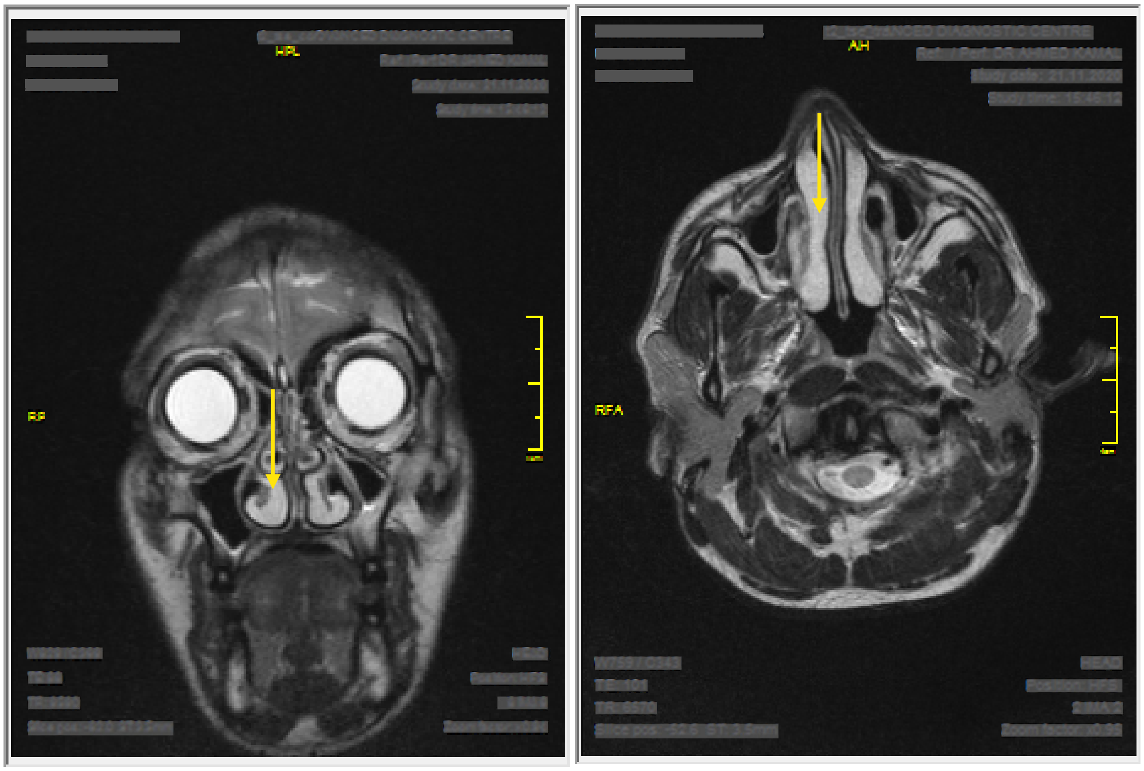

Figure S2. MRI images of individual IV-3 to document signs of PCD. In the left panel, the yellow arrow points to thick nasal mucosae as a sign of mild manifestation of PCD. However, the sinuses of the patient are free (yellow arrow in the right panel).

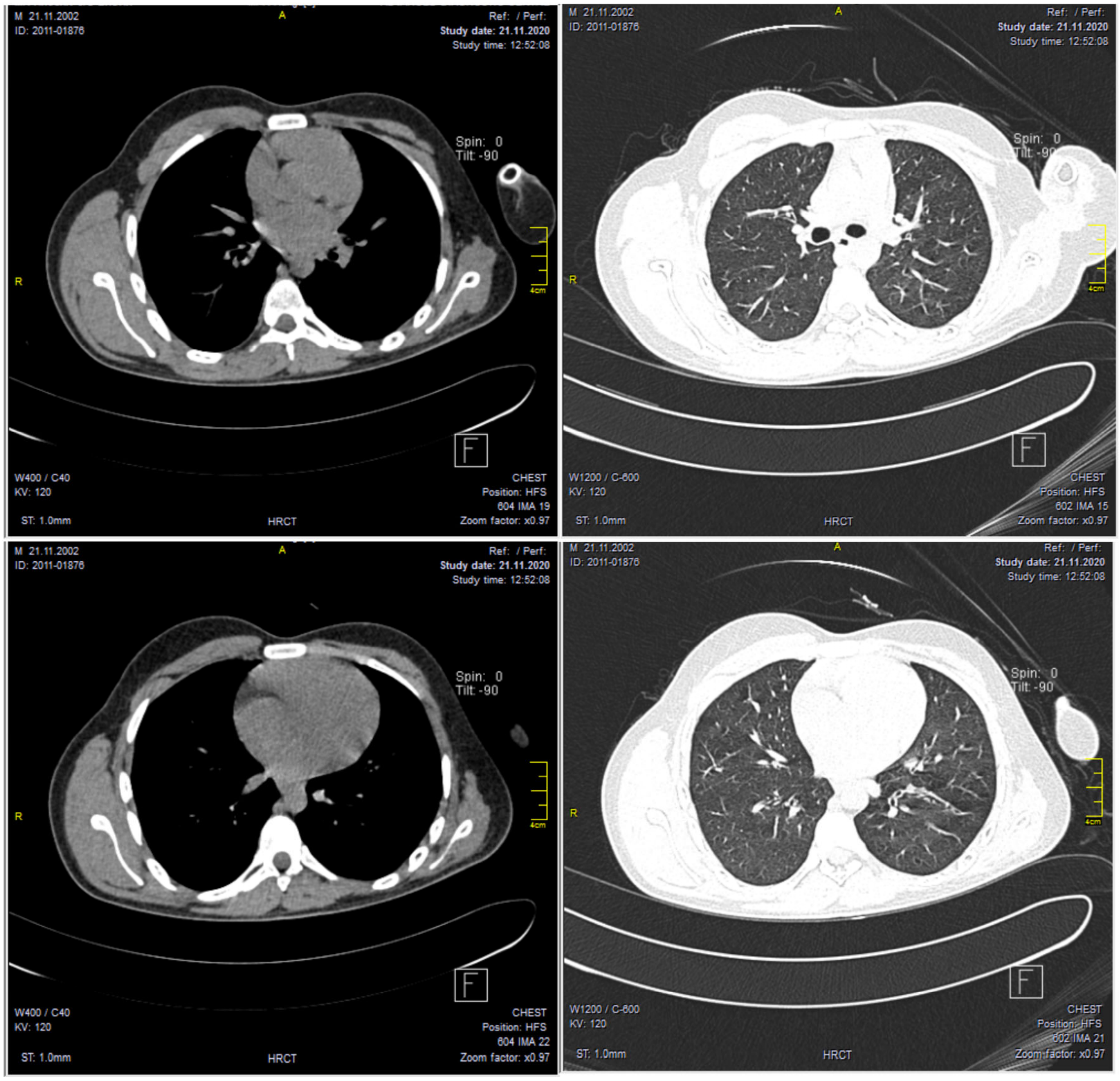

Figure S3. Thoracic CT scan of individual IV-3. The lungs appear clear and normal, no honey combing, nodule, collapse or mass lesion. Heart, abdomen and osseous structures are normal. Mediastinal adenopathy and pleural or pericardial effusion are not present.

Table S1. Highest ranking homozygous, X-linked and compound heterozygous variants detected by WES.

Chr.	Gene	Variant	Mutation Taster	CADD PHRED	Polyphen-2 HDIV(v2.2,2)	dbSNP	ClinVar	gnomAD v2.1.1 allele frequency	Variant nature	Conclusion
9	*AKNA*	NM_030767.4: c.2737delG p.(Glu913Argfs*42) homozygous	Disease causing	26.5	NA	rs368704395	Absent	0.00003416 9 heterozygotes of African ethnicity 0 homozygotes	LOF	Plausible candidate
X	*TSPYL2*	NM_022117.3: c.2033A>C p.(Gln678Pro) hemizygous	Polymorphism (76)	5.122	0.01	rs781788384	Absent	0.0002235 21 heterozygotes 20 hemizygotes	Missense	Excluded
15	*C15orf40*	NM_001160115.1: c.82A>C, p.(Met28Leu) compound heterozygous	Polymorphism (32)	22.2	0.012	rs140417286	Absent	0.0003486 96 heterozygotes 0 homozygotes	Missense	Excluded
		NM_001160115.1: c.73T>C, (p.Cys25Arg) compound heterozygous	Polymorphism (NA)	8.391	NA	rs4842860	Absent	0.6244 170578 heterozygotes 54701 homozygotes	Missense	Excluded

Note: NA, Not Available, LOF**,** Loss-of-function
